# Multitechnique approach for peri-mitral flutter: A case report of combining direct vein of Marshall ethanol infusion and alpha loop ablation

**DOI:** 10.1016/j.jccase.2025.05.009

**Published:** 2025-06-14

**Authors:** Taishi Fukushima, Yoshihiro Sobue, Eiichi Watanabe, Hideo Izawa

**Affiliations:** a Department of Cardiology, Fujita Health University Bantane Hospital, Nagoya, Aichi, Japan; b Department of Cardiology, Fujita Health University Hospital, Toyoake, Aichi, Japan

**Keywords:** Case report, Peri-mitral flutter, Alpha loop ablation, Epicardial connection

## Abstract

The management of peri-mitral flutter often necessitates a vein of Marshall (VOM) ethanol infusion (EI) and radiofrequency ablation within the coronary sinus (CS). These procedures can be technically demanding due to the anatomical constraints and require a nuanced understanding of catheter techniques. We report a patient who experienced dual tachycardias involving a peri-mitral flutter and roof-dependent atrial tachycardia after cryoballoon pulmonary vein isolation. Since linear ablation of the lateral mitral isthmus failed to eliminate the tachycardia, the involvement of epicardial structures such as the VOM and CS was suggested. Attempts at a VOM-EI using a catheter with a lumen succeeded in delivering ethanol but failed to terminate the arrhythmia. Standard techniques for catheter insertion into the CS were unsuccessful. By employing an alpha loop catheter configuration via the right femoral vein, a successful catheter insertion was achieved, enabling the ablation and immediate termination of the tachycardia. This case underscores the importance of employing innovative techniques, such as the alpha loop method and the direct VOM-EI via small-lumen catheters, in cases where standard approaches are insufficient. These methods provide viable alternatives for achieving successful outcomes in peri-mitral flutter management, especially when epicardial connections complicate the procedure.

**Learning objective:**

When standard catheter insertion into the coronary sinus is unsuccessful via the femoral vein, employing an alpha loop configuration can be an effective alternative.

Direct ethanol infusion through a catheter with an inner lumen, rather than using over-the-wire balloon techniques, can be a viable option for a vein of Marshall ethanol infusion in anatomically challenging cases.

## Introduction

Atrial tachycardia (AT) is a frequent complication following catheter ablation of atrial fibrillation (AF), posing significant management challenges. Peri-mitral flutter, which accounts for approximately 13 %–34 % of post-ablation AT episodes, is particularly notable due to its complex mechanisms and challenging anatomical context [1,2]. Achieving a complete mitral isthmus block-line is often complicated by factors such as the anatomical intricacy of the surrounding structures, including the myocardial sleeves of the coronary sinus (CS) and vein of Marshall (VOM), as well as the heat sink effect created by the CS blood pool [3]. In addition, catheter cannulation of the CS or VOM can be challenging due to anatomical limitations. We present a challenging peri-mitral flutter case after cryoballoon ablation of persistent AF, which required not only creating an endocardial mitral isthmus block-line but also an ethanol infusion into the VOM (VOM-EI) and radiofrequency ablation in the CS using an alpha loop configuration of the ablation catheter.

## Case report

A 70-year-old Japanese male with a history of persistent AF was referred to our hospital. Despite undergoing a prior pulmonary vein (PV) isolation using a cryoballoon (Medtronic, Dublin, Ireland) and cavo-tricuspid isthmus linear ablation, he experienced an early recurrence of persistent AF. Eight months later, a second session was performed.

**Fig. 1:**
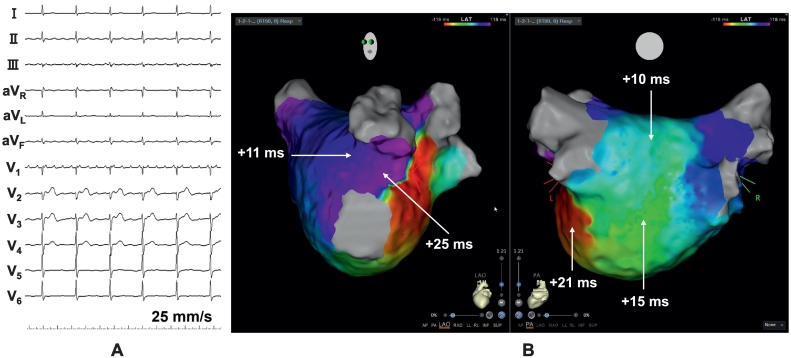
(A) Twelve‑lead electrocardiogram of atrial tachycardia. (B) The numbers represent the postpacing interval minus tachycardia cycle length at each site (white arrow).

**Fig. 2:**
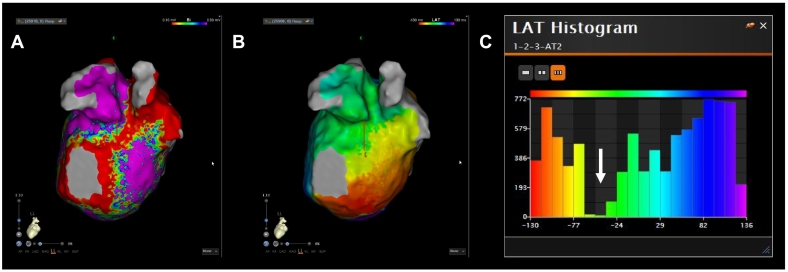
(A) The left atrial voltage map after radiofrequency ablation of the lateral mitral isthmus line shows the line of block. (B) The left atrial local activation time (LAT) map shows atrial tachycardia rotating counterclockwise around the mitral annulus. (C) But the LAT histogram reveals missing temporal segments (white arrow).

**Fig. 3:**
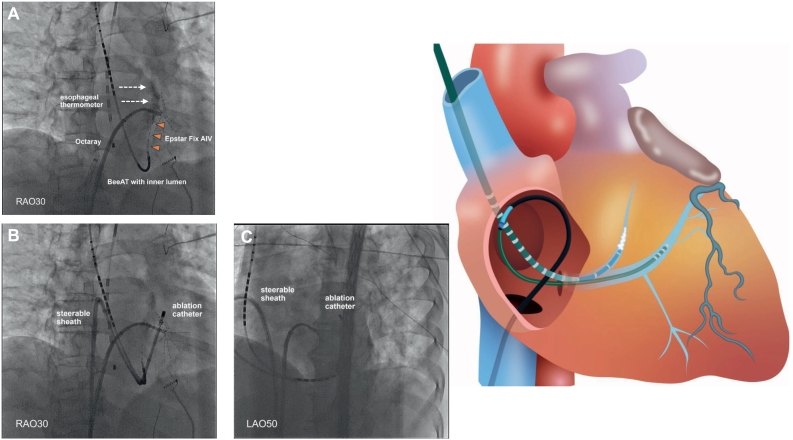
(A) The EPstar Fix AIV catheter was sufficiently wedged in the vein of Marshall (VOM). Note that the contrast agent was pooling (dotted arrow). Direct ethanol infusion via EPstar Fix AIV catheter was performed. (B, C) Successful ablation catheter insertion into the coronary sinus with an alpha loop configuration.

A 20-pole 3-site (CS: 8 poles; right atrium: 8; and superior vena cava: 4) mapping catheter with an inner lumen (BeeAT with inner lumen, Japan Lifeline, Tokyo, Japan) was positioned at the CS ostium due to a Vieussens valve. An irrigated ablation catheter (THERMOCOOL SMARTTOUCH SF Catheter, Biosense Webster, Irvine, CA, USA) and 48-electrode high-resolution mapping catheter (Octaray, Biosense Webster) were advanced into the left atrium (LA). Following cardioversion, during sinus rhythm, LA mapping using a CARTO3 mapping system (Biosense Webster) and Octaray catheter revealed residual potentials on the carina of the right PV and posterior wall of the right inferior PV. During LA mapping, an AT with a cycle length of 240 ms was observed (Fig. 1A). AT mapping and a postpacing interval (PPI) analysis suggested a dual tachycardia involving peri-mitral and roof-dependent ATs (Fig. 1B), with a common pathway located at the left PV (LPV) anterior carina and lateral mitral isthmus. Linear radiofrequency ablation with 40 W, utilizing a point-by-point technique targeting an ablation index of 500, was performed from the mitral valve 3 o'clock position to the anterior region of the left inferior PV. That successfully terminated the tachycardia (Online Fig. S1). The CS activation sequence, after advancing the CS catheter using the guide wire to the distal CS, indicated a mitral isthmus block during distal CS pacing (Online Fig. S2). In addition, differential pacing from both distal and proximal CS electrodes confirmed bidirectional conduction block across the mitral isthmus. Subsequently, a right PV re-isolation and roof/bottom line ablation were added for the roof-dependent AT, resulting in electrical isolation of the LA posterior wall. During repeat LA mapping, the AT reappeared. LA mapping suggested the mitral block-line was completed (Fig. 2A). Similar to the beginning, the tachycardia rotated counterclockwise around the mitral annulus (Fig. 2B). But the local activation time histogram revealed a missing temporal segment (Fig. 2C), suggesting VOM involvement. Therefore, a 2.7 Fr decapolar electrode catheter (EPstar fixed AIV, Japan Lifeline) was inserted into the VOM through the BeeAT with inner lumen after contrast enhancement (Online Fig. S3A, B). The EPstar Fix AIV catheter could not be inserted to the distal portion of VOM due to the small vessel size. The PPI in the VOM was 278 ms, which was 27 ms longer than the tachycardia cycle length (TCL) of 251 ms (Online Fig. S4). The PPI in the VOM was not completely consistent with the TCL, but the value suggested it was close to the circuit. The inability to pace from the VOM's distal portion was considered the cause of the discrepancy. It was confirmed the EPstar Fix AIV catheter was wedged in the VOM based on the contrast imaging findings. The VOM-EI was delivered through the EPstar Fix AIV catheter's lumen, not an over-the-wire balloon (Fig. 3A). After pulling the AIV catheter back slightly, contrast injection via the catheter lumen confirmed contrast pooling in the VOM, indicating adequate engagement. A second VOM-EI was then performed, but the tachycardia persisted. It was considered that ablation in the CS was needed, however, the ablation catheter could not be advanced using the standard approach. After trial and error, the ablation catheter was successfully inserted into the CS by creating an alpha loop configuration (Fig. 3B, C). The PPI in the CS on the opposite side of the mitral-line coincided with the TCL (Online Fig. S5A, B). At that site, fragmented potentials were recorded by the ablation catheter's distal electrodes (Online Fig. S5B). Ablation at that site immediately terminated the tachycardia (Online Fig. S6). Despite the optimal ablation site, adequate radiofrequency applications could not be achieved because of a marked rise in impedance. Therefore, by manually increasing the ablation catheter tip's irrigation flow rate to 30 mL/min, adequate radiofrequency applications with 30 W for 17 s (ablation index of 358, average contact force of 10 g, initial impedance of 139 Ω) were possible. Thereafter, the tachycardia could no longer be induced. The final LA mapping exhibited a vast mitral isthmus block area (Online Fig. S7).

## Discussion

This case highlights a specific solution to technical challenges in the management of peri-mitral flutter—namely, when advancement of the ablation catheter into the CS or the balloon catheter into the vein of VOM is limited by various anatomical barriers. By utilizing an alpha loop configuration and performing direct VOM-EI without a balloon catheter, we were able to overcome these difficulties and successfully terminate the peri-mitral flutter. These techniques may offer useful alternatives in cases where standard approaches are not feasible.

A VOM-EI was performed prior to radiofrequency ablation within the CS to avoid potential thermal injury or edema at the VOM ostium, which might impair subsequent ethanol delivery [4]. Takigawa et al. suggested that initiating treatment with VOM-EI followed by radiofrequency ablation significantly reduces AT recurrence and improves mitral isthmus block durability compared with radiofrequency ablation alone, resulting in a 65 % reduction in recurrence risk at one year [5]. Gillis et al. likewise demonstrated that initiating VOM-EI significantly improves mitral isthmus block success rates compared to performing radiofrequency ablation first (94 % vs. 63 %) [4]. In addition, patients without an anatomically visible VOM exhibited a markedly higher success rate of posterior mitral isthmus ablation, indirectly suggesting that the presence of the VOM contributes to persistent conduction across the isthmus [6]. Typically, a VOM-EI is performed by occluding the VOM using an over-the-wire balloon and injecting ethanol through the wire's lumen. However, in this case, cannulating the VOM was extremely challenging, and wiring and delivering an over-the-wire balloon was deemed to be difficult. Therefore, it was confirmed that the EPstar Fix AIV catheter was successfully wedged, and ethanol was directly injected into the VOM through this catheter. No complications associated with this procedure were observed, and the LA voltage map exhibited a well-defined no-voltage area (Online Fig. S7).

Moreover, in this case, the insertion of the ablation catheter into the CS was extremely challenging. Insertion of an ablation catheter into the CS is occasionally difficult due to a Thebesian valve, Chiari network, Vieussens valve, or the size or angle of the vessels. In such cases, an internal jugular vein or subclavian vein approach can sometimes be useful; however, the VOM catheter had already been inserted through the internal jugular vein and was unavailable for use. Therefore, it was necessary to insert the CS catheter via the femoral vein. In this case, the ablation catheter could not be inserted into the CS using the standard approach. Since an alpha loop is sometimes created when inserting electrophysiological catheters into the CS, this technique was applied, and the ablation catheter was shaped into an alpha loop, which successfully allowed its insertion into the CS. By inserting the catheter into the CS with an alpha loop configuration, it became easier to achieve contact on the lesser curvature side, namely the myocardial side. Forming an alpha loop with the ablation catheter alone is technically challenging; however, the use of a steerable sheath enables stable loop formation and controlled advancement into the CS. Moreover, the use of contact force-sensing catheters may contribute to enhanced procedural safety during catheter manipulation in anatomically complex regions. To our knowledge, this is the first report that specifically focuses on the use of an alpha loop configuration to facilitate catheter insertion into the CS during electrophysiological procedures. This method was considered a useful option when catheter insertion into the CS is challenging with standard approaches.

## Conclusion

This case highlights the need for tailored strategies in managing peri-mitral flutter, particularly in anatomically complex scenarios. Techniques such as a VOM-EI through small-lumen catheters and an alpha loop catheter configuration for inserting CS catheters provide essential alternatives to standard methods. Incorporating these approaches into clinical practice may enhance treatment outcomes in challenging AT cases.

## Consent

The authors confirm that written consent for submission and publication of this case report including the images and associated text has been obtained from the patient in line with COPE guidance.

## Funding

None declared.

## Declaration of competing interest

The authors state that they have no conflict of interest regarding this paper.

## Appendix A Supplementary data

Online Fig. S1 The atrial tachycardia terminated during the radiofrequency ablation of lateral mitral isthmus. Online Fig. S2 Atrial potentials recorded with CS electrodes show proximal-to-distal propagation (orange arrow). This indicates a mitral isthmus block. Online Fig. S3 (A) The contrast enhancement of the vein of Marshall (VOM, white arrowhead) through BeeAT with inner lumen catheter. (B) EPstar Fix AIV catheter (orange arrowhead) was inserted into the VOM through BeeAT with inner lumen catheter. Online Fig. S4 The postpacing interval in the vein of Marshall (VOM) was slightly longer than the tachycardia cycle length. Online Fig. S5 (A) The numbers represent the postpacing interval minus tachycardia cycle length at each site (orange arrow). (B) The distal electrode of ablation catheter recorded the fragmented potentials (black arrow). At this site, the postpacing interval was almost perfectly consistent with the tachycardia cycle length. Online Fig. S6 The tachycardia was immediately terminated by radiofrequency ablation within the CS. Online Fig. S7 The post voltage map shows a large no voltage area around the lateral mitral isthmus. Image 1
