# Supplementary material for: Multitechnique approach for peri-mitral flutter: A case report of combining direct vein of Marshall ethanol infusion and alpha loop ablation
Source: J Cardiol Cases. 2025 Jun 14;32(3):142–5. doi: 10.1016/j.jccase.2025.05.009 (PMC12432514; doi:10.1016/j.jccase.2025.05.009)
Supplement: Supplementary file 1 — Online Fig. S1 The atrial tachycardia terminated during the radiofrequency ablation of lateral mitral isthmus. Online Fig. S2 Atrial potentials recorded with CS electrodes show proximal-to-distal propagation (orange arrow). This indicates a mitral isthmus block. Online Fig. S3 (A) The contrast enhancement of the vein of Marshall (VOM, white arrowhead) through BeeAT with inner lumen catheter. (B) EPstar Fix AIV catheter (orange arrowhead) was inserted into the VOM through BeeAT with inner lumen catheter. Online Fig. S4 The postpacing interval in the vein of Marshall (VOM) was slightly longer than the tachycardia cycle length. Online Fig. S5 (A) The numbers represent the postpacing interval minus tachycardia cycle length at each site (orange arrow). (B) The distal electrode of ablation catheter recorded the fragmented potentials (black arrow). At this site, the postpacing interval was almost perfectly consistent with the tachycardia cycle length. Online Fig. S6 The tachycardia was immediately terminated by radiofrequency ablation within the CS. Online Fig. S7 The post voltage map shows a large no voltage area around the lateral mitral isthmus. [file mmc1.pptx]

## Slide 1
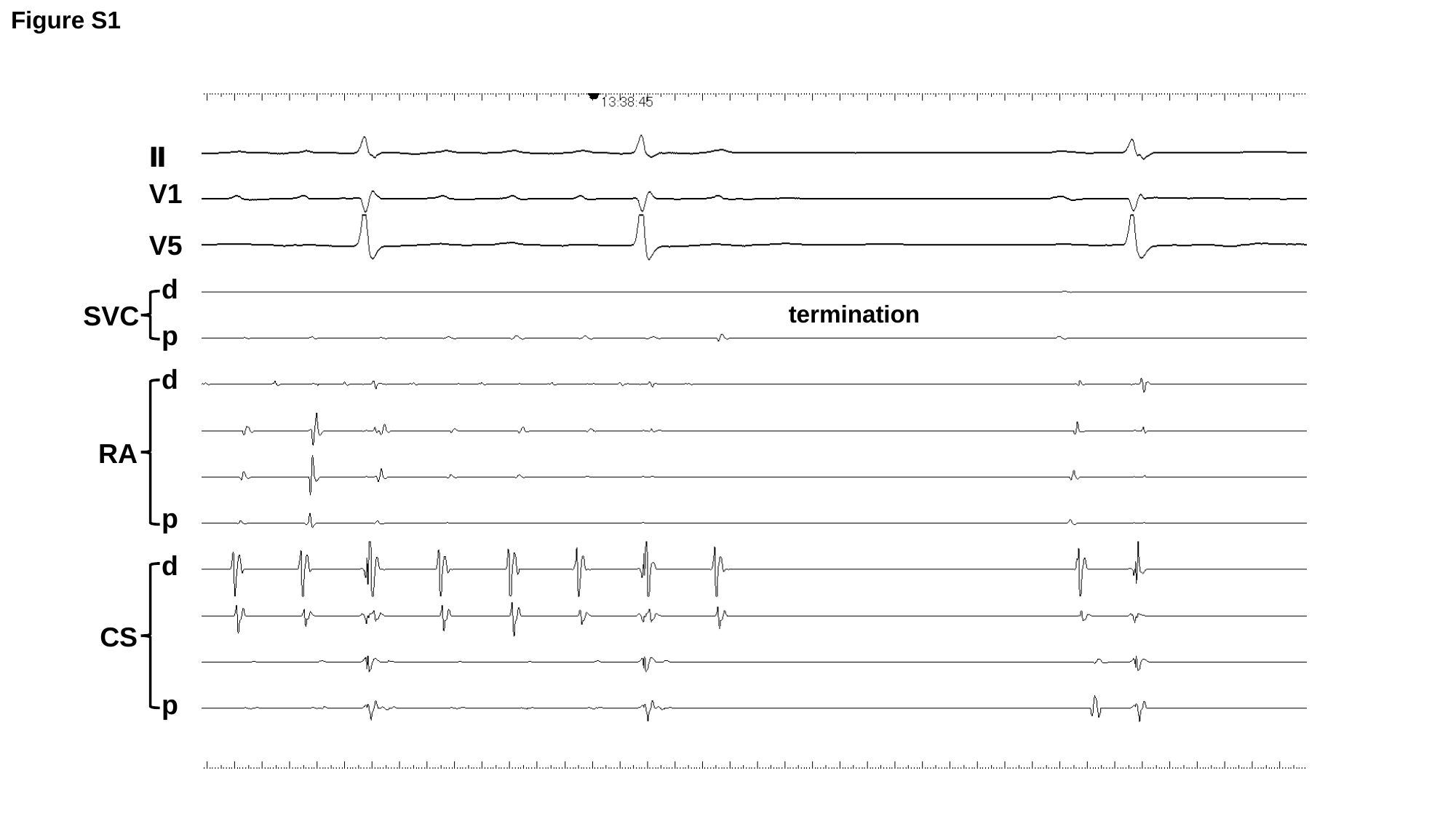

# Figure S1
Ⅱ
V1
V5
d
SVC
termination
p
d
RA
p
d
CS
p

## Slide 2
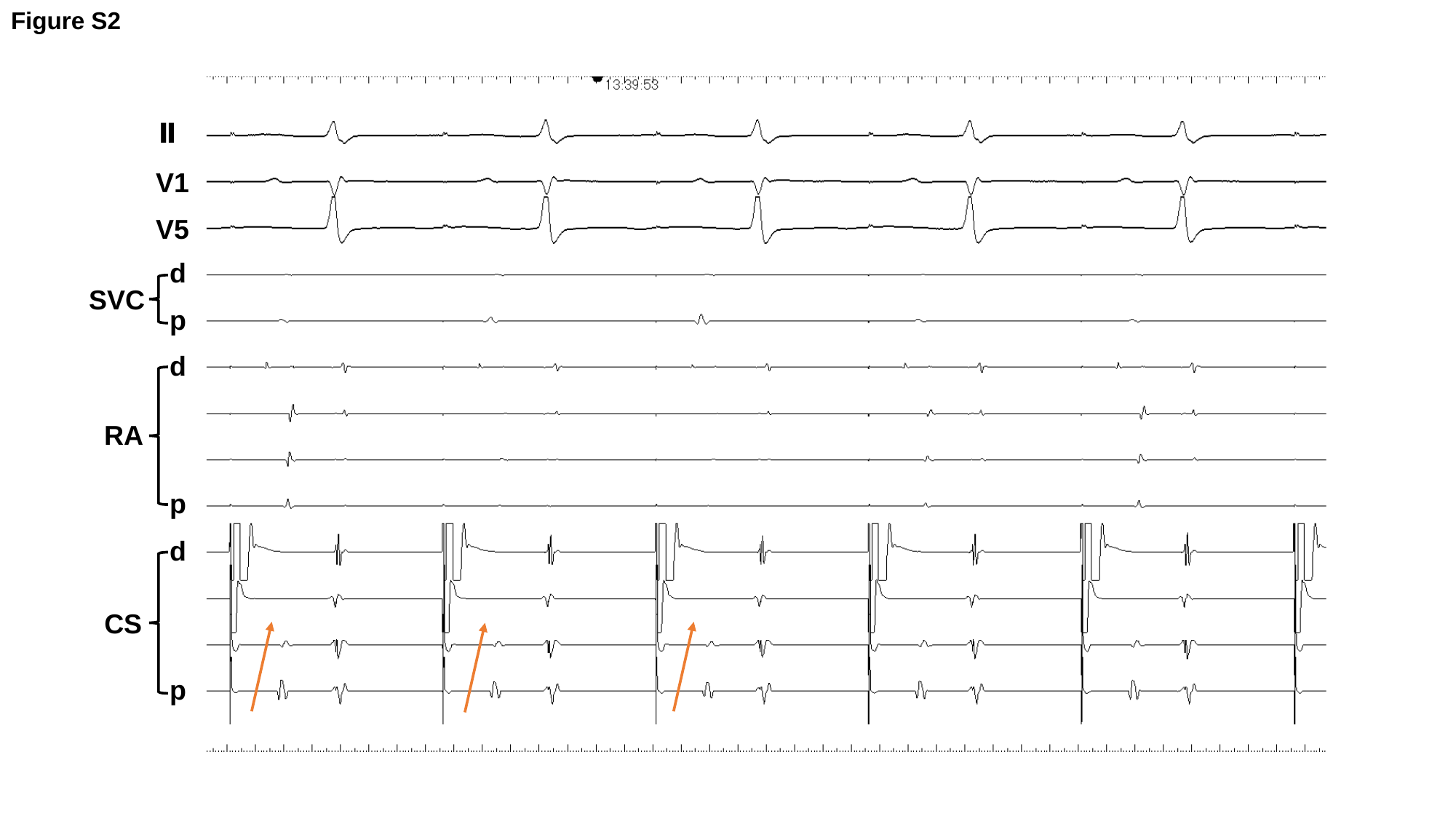

# Figure S2
Ⅱ
V1
V5
d
SVC
p
d
RA
p
d
CS
p

## Slide 3
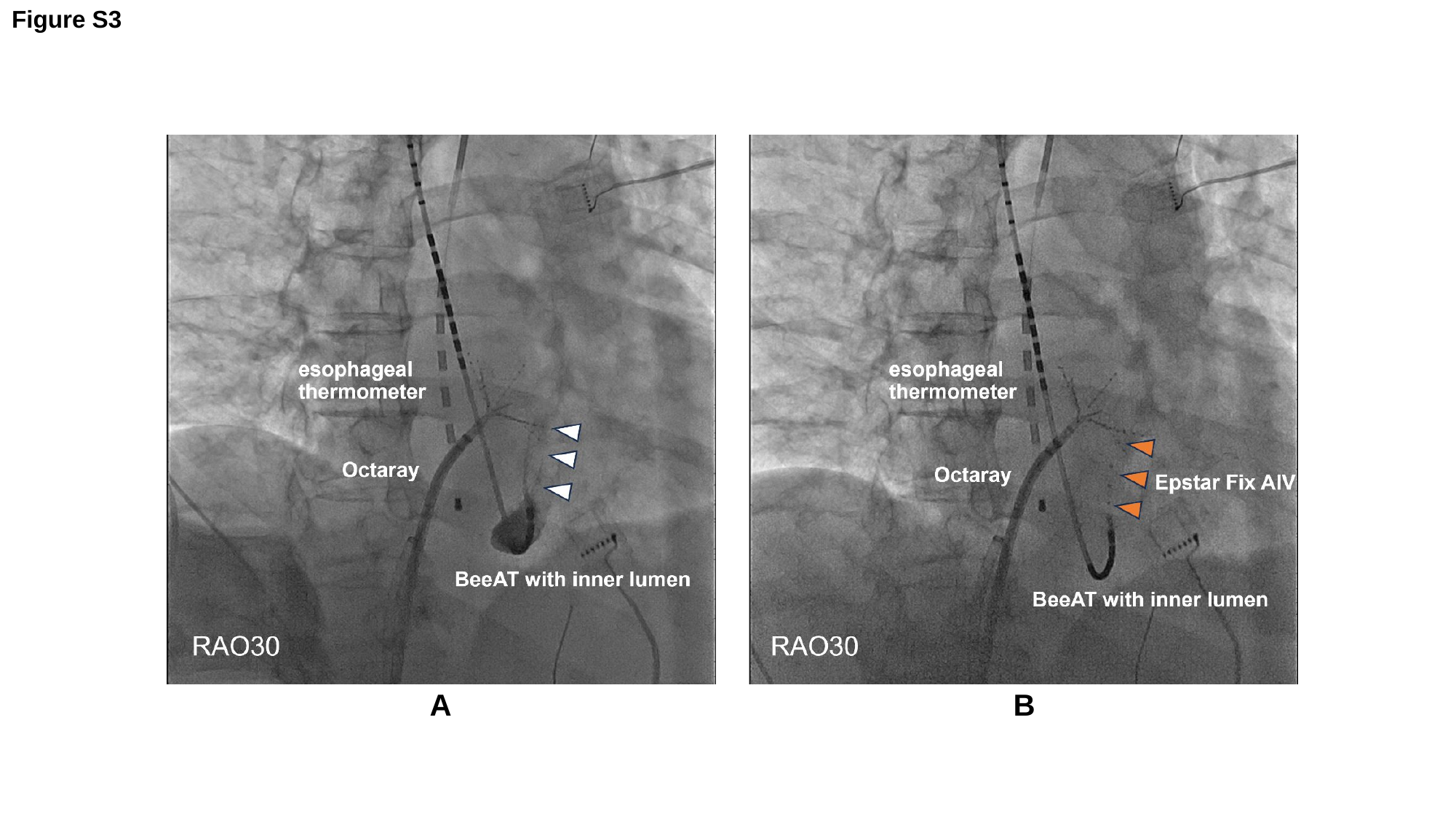

# Figure S3
A
B

## Slide 4
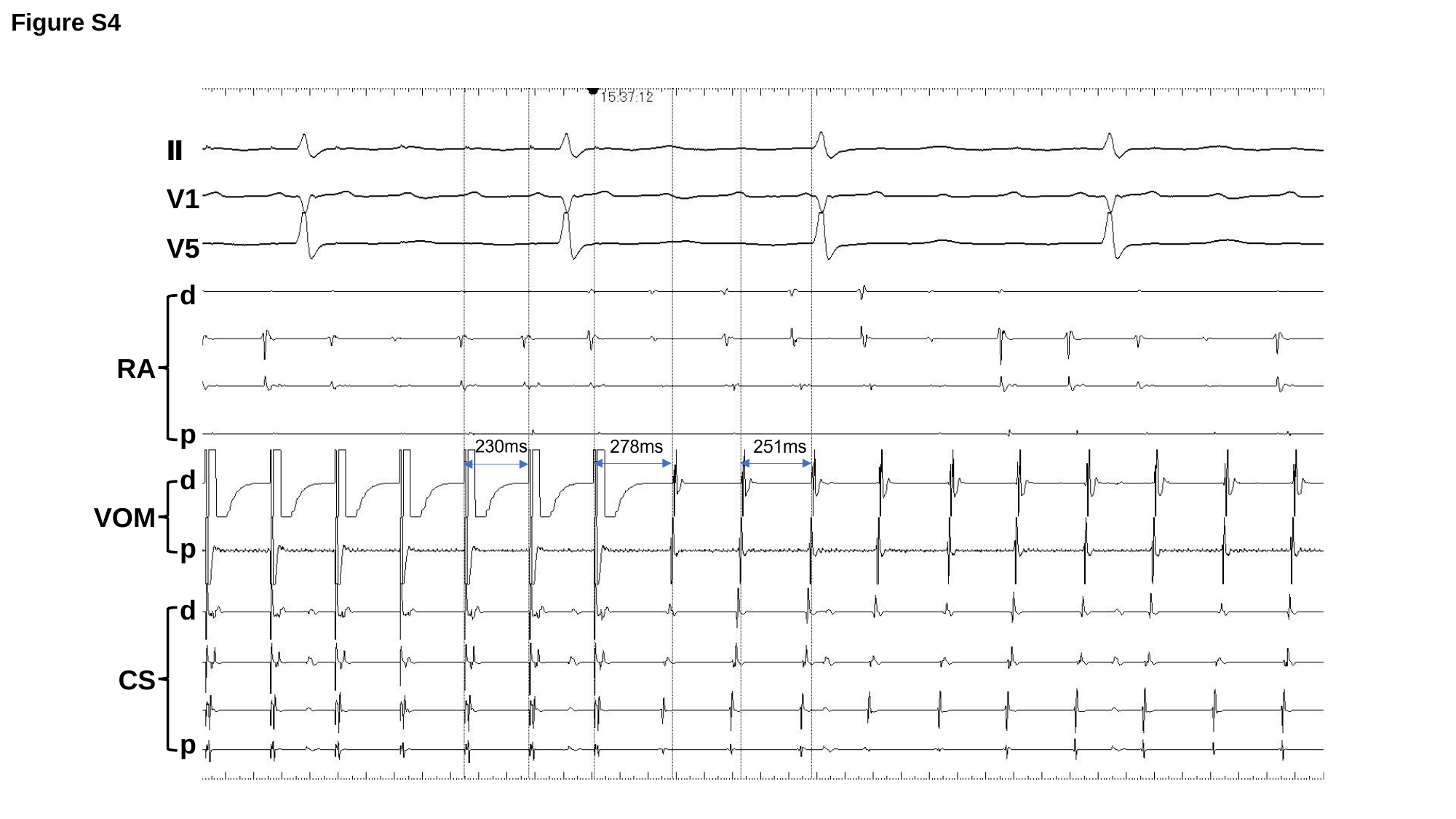

# Figure S4
Ⅱ
V1
V5
d
RA
p
d
VOM
p
d
CS
p

## Slide 5
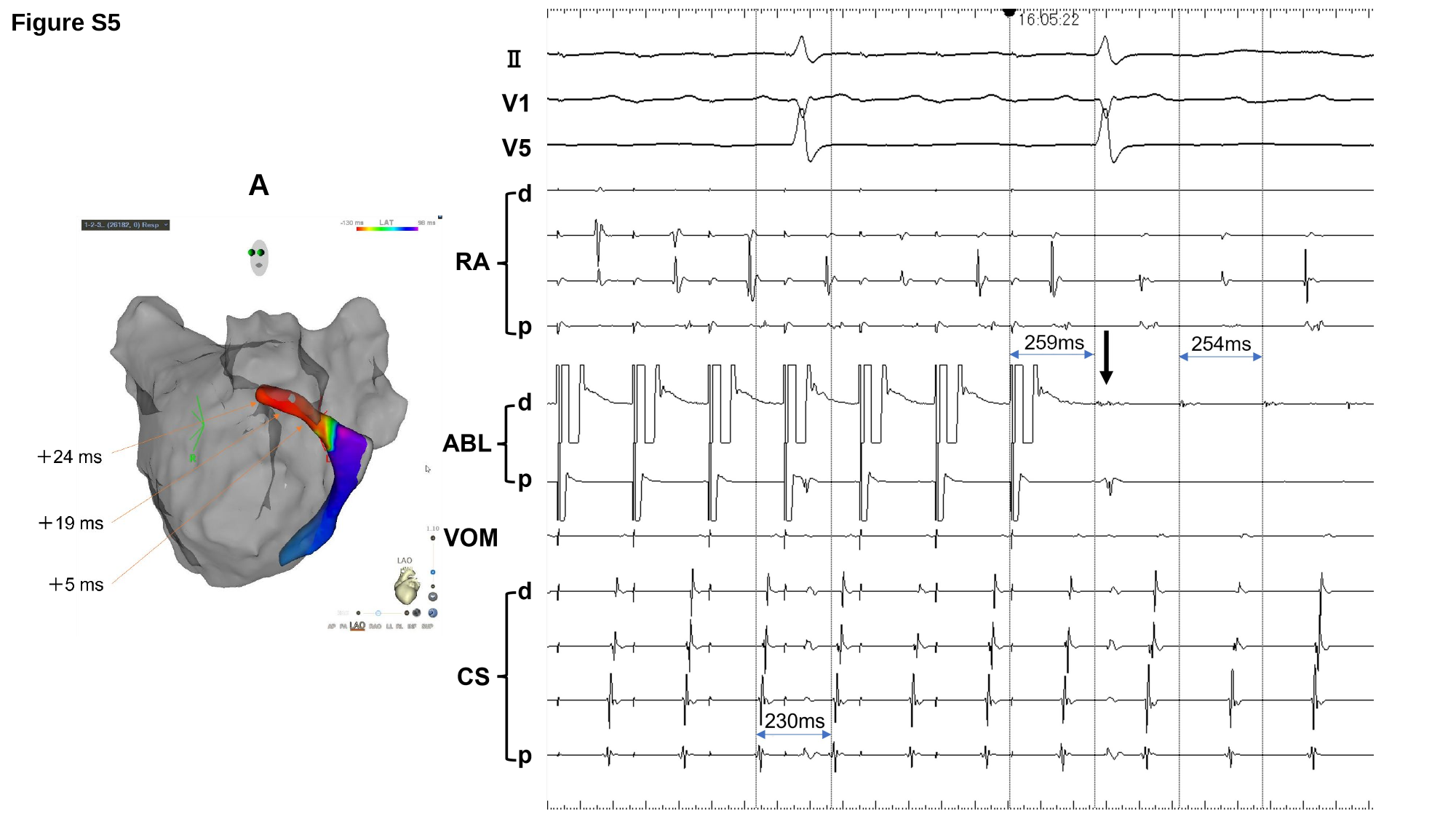

B
# Figure S5
A

## Slide 6
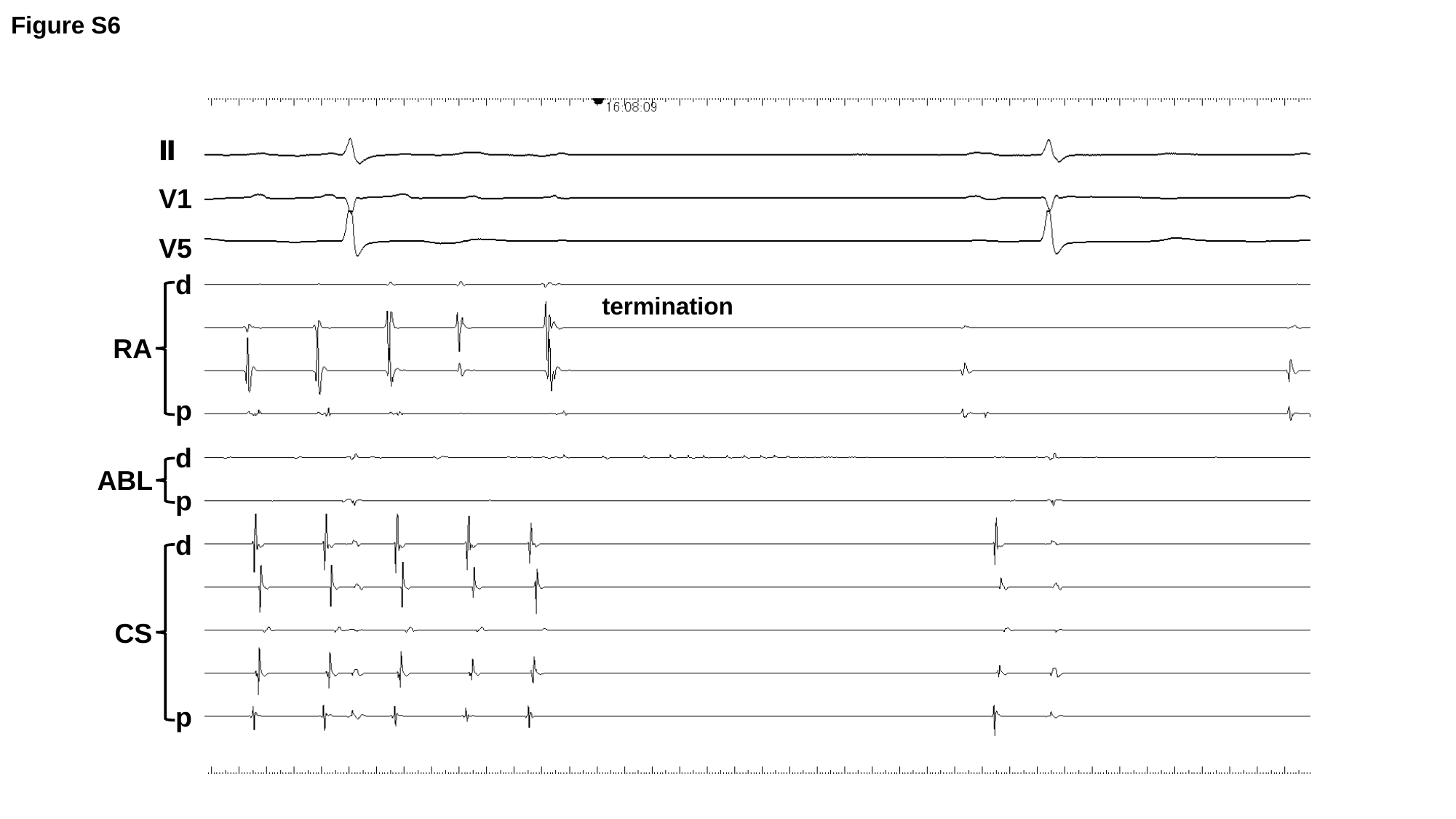

# Figure S6
Ⅱ
V1
V5
d
termination
RA
p
d
ABL
p
d
CS
p

## Slide 7
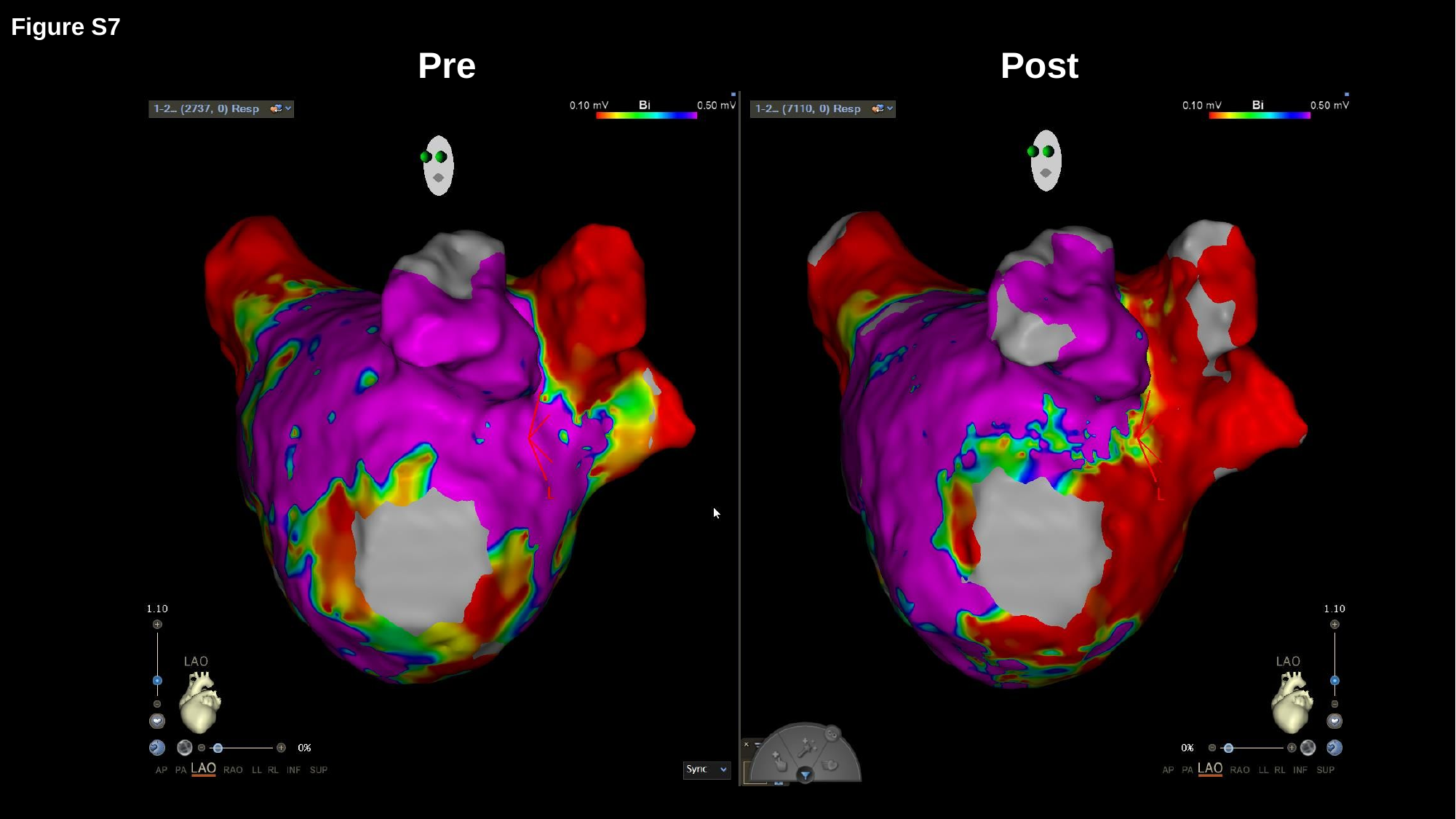

# Figure S7
Pre
Post
